# Fever detection in under 5 children in a tertiary health facility using the infrared tympanic thermometer in the oral mode

**DOI:** 10.1186/1824-7288-37-8

**Published:** 2011-01-22

**Authors:** Benedict O Edelu, Ngozi C Ojinnaka, Anthony N Ikefuna

**Affiliations:** 1 Department of Pediatrics, University of Nigeria Teaching Hospital, Ituku/Ozalla, Enugu, Nigeria

## Abstract

### Background

Monitoring of body temperature is an important clinical procedure in the care of sick children, especially the under-5 children, as many disease conditions present with fever. The oral mercury-in-glass thermometer which has relatively good accuracy cannot be used in children less than 5 years because it requires their cooperation.

### Objective

This study was aimed at using the infrared tympanic thermometer (IRTT) in oral mode to measure temperature in febrile and afebrile children less than 5 years.

### Methods

Rectal and tympanic temperatures were measured consecutively in 400 febrile and 400 afebrile under-5 children matched for age, using the mercury-in-glass thermometer and the IRTT in oral mode respectively.

### Results

In the febrile children, the mean tympanic temperature was 38.6 ± 0.9°C, while the mean rectal temperature was 39.0 ± 0.8°C. In the afebrile group, the mean tympanic temperature was 37.0 ± 0.4°C, while the mean rectal temperature was 37.4 ± 0.3°C. The mean difference between rectal and tympanic temperatures in both groups was statistically significant. There was good correlation between the two temperatures. The tympanic thermometer used in the oral mode had a sensitivity of 87.3% and a specificity of 96.5%.

### Conclusion

The IRTT (oral mode) may not be reliable in estimating 'core' body temperature in children under the age of five years, but with a fairly good sensitivity and specificity, as well as its other advantages such as short duration of measurement, convenience and safety, it is a useful instrument for screening children with fever in a busy setup.

## Background

The best site to measure 'core' temperature is the temperature regulating centre situated in the hypothalamus, but since this is not feasible, body sites that most closely approximate the 'core' temperature provide the most accurate readings [1]. The pulmonary artery, oesophagus, trachea, nasopharynx and bladder have all been used in anaesthetized patients [2]. However, access to these sites require invasive procedures and are not feasible for routine clinical use, hence the use of rectal temperature as the 'gold standard' in most clinical settings [3,4]. Apart from the rectum, the oral cavity and axilla have traditionally been used to take temperature in children, using mercury- in -glass thermometer. In recent years, however, the use of mercury thermometers has been discontinued by several countries in Europe and some states in United States due to the risk of mercury poisoning.

Studies [5,6] have shown that oral temperature values closely approximated that obtained by rectal route and so the former can be used in the estimation of core body temperature. The axillary temperature, on the other hand varied widely with rectal temperature, except in neonates [7,8]. However, oral temperature measurement cannot be used effectively in children below 5 years of age due to lack of cooperation and the difficulty in ensuring an appropriate mouth seal to get a good reading. Unfortunately, rectal thermometry has been resented by many children and their parents [3,9] leaving axillary thermometry as the only option despite its poor value [2,7].

The infrared tympanic thermometer (IRTT) has, therefore, come as a ready alternative in this age group. The tympanic membrane shares blood supply with the hypothalamus and it is thought by some to be the ideal location for core body temperature measurement [3,9]. The IRTT has the advantage of speed and convenience [10,11] as well as the unique feature of generating readings in different modes (tympanic, oral or rectal), depending on the thermometer brand. The infrared tympanic thermometer used in any mode, implies that the temperatures displayed are the particular mode equivalent of the actual readings. The manufacturers use a numeric constant known as offset to generate readings that the clinicians are more familiar with. This may offer an advantage in the sense that most Physicians in our environment are more conversant with fever cut-off for oral, axillary and rectal temperature readings and so can interpret readings better. Some studies [7,9,12,13] have questioned the accuracy of the tympanic thermometry, while others [2-5,14-17] supported its use. Despite this inconsistency, the infrared tympanic thermometry can be seen in some clinics in Nigeria.

This study was aimed at using the infrared tympanic thermometer in oral mode to evaluate temperatures in children less than 5 years, in who direct oral temperature measurement is difficult. The sensitivity, specificity and predictive values of the instrument were also determined.

## Materials and methods

Four hundred febrile children less than 5 years of age (birth - 59 months) were recruited from the children's outpatient clinic and children's emergency room at the University of Nigeria Teaching Hospital, Enugu, Nigeria. They were matched for age with 400 afebrile children from the well baby and immunization clinics. The children were stratified based on their ages into neonates, infants, 12 - 23 months, 24 - 35 months, 36 - 47 months and 48 - 59 months.

Fever in this study was defined as rectal temperatures of ≥ 37.6°C in neonates and ≥ 38.0°C in the older children, tympanic (oral mode) temperature of ≥ 37.6°C for neonates and ≥ 37.8 for older children.

The afebrile children had no complaints of fever, no history of immunization in the preceding 7 days and normal physical findings on examination. Children with suppurative otitis media, or otitis externa were excluded.

Approval from the research and ethics committee of the hospital was obtained. Written consents were also obtained from the parents or guardians.

Before taking any temperature, history was taken and necessary data such as age, sex, presenting complaints, if any, were obtained. This was followed by a general examination and auroscopy to ensure that the tympanic membranes were intact and normal. The rectal mercury-in-glass thermometer was lubricated with water - soluble lubricant and inserted into the rectum to a depth of 2 - 3 cm in neonates and 5 - 6 cm in older children and left for 3 minutes and 5 minutes respectively in neonates and older children before removal for reading.

The tympanic temperature was taken immediately the rectal thermometer was removed. In taking the tympanic temperature, the ear was pulled straight back in infants and in older children it was pulled up and back with the child still in lying position. This was to expose the tympanum. The probe was then inserted into the ear and left till there was a beep signifying the end of measurement. (This procedure conformed to the manufacturer's instruction and took only a few seconds.). The probe cover was changed before taking another temperature. Temperature was taken once from one ear only as studies [9,18] have demonstrated very good correlation between the two ears. Temperature was taken from the right ear in all the subjects for uniformity and convenience. All the children with fever were investigated and treated as appropriate for each child.

The tympanic thermometer used was OMRON^® ^instant ear thermometer model MC - 509 N. This ear thermometer takes 12 temperature scans within one second and then displays the highest temperature. It has a measuring range of 32.0°C to 42.2°C, with a laboratory accuracy of ±0.2°C. It measures in the oral mode, implying that measured temperature is converted to oral temperature equivalent before display. To standardize the mercury-in-glass thermometers, the thermometers were placed in warm water bath and ensured that all readings were the same before use each day. Each rectal mercury-in-glass thermometer was used only once each day and properly disinfected afterward. The infrared tympanic thermometers were compared with one another each day by taking temperature reading from a particular ear before the start of daily measurements to ensure that readings were similar. The two thermometers used showed similar readings throughout the study.

The data were analyzed with the computer, using the SPSS-15 software. Rectal temperature was used as a reference standard to compare tympanic temperature. The mean differences between rectal and tympanic temperatures were compared using the Student's t test. A p-value of less than 0.05 was considered statistically significant. The relationship between the two methods of temperature measurement was determined using the Pearson's correlation coefficient at 99% confidence limit. The sensitivity, specificity, positive and negative predictive values were also calculated.

## Results

A total of eight hundred children under the age of 5 years were studied. This consisted of 400 febrile and 400 healthy, afebrile children. There were 429 males and 371 females, giving a male: female ratio of 1.2:1. The age distribution was compatible in both groups (p = 0.35). See Table 1.

**Table 1:** Age and sex distribution of the total population studied

Age group(Months)	n	Rectal temp. Mean (SD)(°C)	Tymp. temp. Mean (SD)(°C)	Recto-Tympanic mean diff (SD)(°C)	t	P
< 1	45	38.3 (0.6)	38.0 (0.8)	0.28 (0.32)	1.843	**0.069**
1 - 11	114	39.0 (0.7)	38.5 (0.7)	0.51 (0.32)	5.791	0.000
12 - 23	84	38.9 (0.7)	38.5 (0.8)	0.46 (0.36)	3.946	0.000
24 - 35	60	39.1 (0.9)	38.8 (1.0)	0.40 (0.43)	2.257	0.026
36 - 47	55	39.1(0.8)	38.8 (0.8)	0.33 (0.36)	2.238	0.027
48 - 59	42	39.3 (1.0)	39.0 (0.9)	0.28 (0.35)	1.336	**0.185**

0 - 59	400	39.0 (0.8)	38.6 (0.9)	0.41 (0.37)	6.962	0.000

The rectal temperature measurements ranged from 38.0 - 41.4°C in the febrile group of children and from 36.4 - 37.9°C in the afebrile group, while the tympanic temperature readings ranged from 36.6 - 40.8°C in the febrile and from 35.7 - 37.9°C in the afebrile groups of children. Tables 2 and 3 compare the mean tympanic and rectal temperatures in the various age groups in febrile and afebrile children respectively. Beyond the neonatal age, the test values (t) showed a decreasing trend in both febrile and afebrile groups. In the afebrile children, all the age groups demonstrated significant differences between the rectal and the tympanic temperatures. (p values ranged from 0.000 - 0.005)

**Table 2:** Comparison of the mean rectal and tympanic temperatures in the febrile children.

Age group (Months)	n	Rectal temp. Mean (SD)(°C)	Tymp. temp. Mean (SD)(°C)	Recto-Tympanic mean diff (SD)(°C)	t	P
< 1	45	38.3 (0.6)	38.0 (0.8)	0.28 (0.32)	1.843	**0.069**
1 - 11	114	39.0 (0.7)	38.5 (0.7)	0.51 (0.32)	5.791	0.000
12 - 23	84	38.9 (0.7)	38.5 (0.8)	0.46 (0.36)	3.946	0.000
24 - 35	60	39.1 (0.9)	38.8 (1.0)	0.40 (0.43)	2.257	0.026
36 - 47	55	39.1(0.8)	38.8 (0.8)	0.33 (0.36)	2.238	0.027
48 - 59	42	39.3 (1.0)	39.0 (0.9)	0.28 (0.35)	1.336	**0.185**

0 - 59	400	39.0 (0.8)	38.6 (0.9)	0.41 (0.37)	6.962	0.000

**Table 3:** Comparison of the mean rectal and tympanic temperatures in the afebrile children.

Age group (Months)	n	Rectal temp Mean (SD)(°C)	Tymp temp Mean (SD)(°C)	Recto-Tympanic mean diff (SD)(°C)	t	p
< 1	45	37.2 (0.2)	36.9 (0.4)	0.27 (0.29)	4.334	0.000
1 - 11	114	37.5 (0.3)	36.8 (0.4)	0.72 (0.34)	15.046	0.000
12 - 23	84	37.5 (0.3)	36.9 (0.4)	0.56 (0.36)	9.814	0.000
24 - 35	60	37.5 (0.4)	37.1 (0.4)	0.40 (0.33)	5.950	0.000
36 - 47	55	37.4 (0.4)	37.2 (0.5)	0.23 (0.39)	2.888	0.005
48 - 59	42	37.4 (0.4)	37.1 (0.5)	0.26 (0.32)	2.867	0.005

0 - 59	400	37.4 (0.3)	37.0 (0.4)	0.47 (0.39)	17.099	0.000

**Figure 1:**
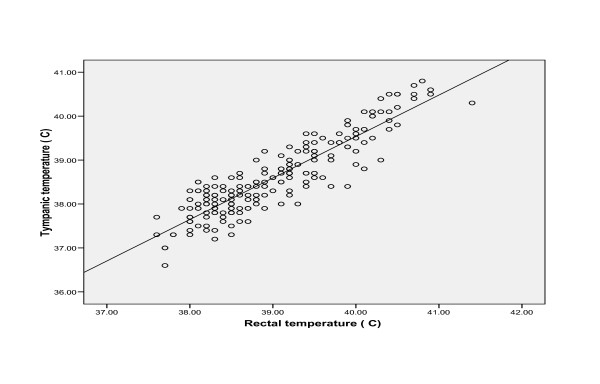
**Scatter diagram showing the relationship between rectal and tympanic temperatures in the febrile children**. r = 0.90

**Figure 2:**
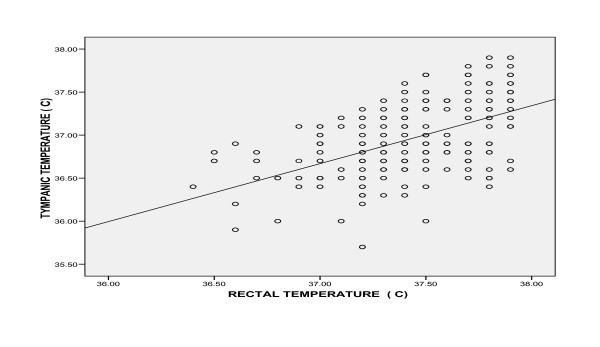
**Scatter diagram showing the relationship between rectal and tympanic temperatures in the afebrile children**. r = 0.52

Using the Pearson's correlation for all the temperatures in the febrile and the afebrile children (figures 1 and 2), the tympanic temperatures showed significant correlation with the rectal temperatures (p < 0.01). The correlation was stronger in the febrile than the afebrile group (r = 0.90 and 0.52 respectively, p < 0.01). The tighter cluster of points in the scatter diagram for the febrile group demonstrated this further.

The overall sensitivity for the IRTT (oral mode) was 87.3% and sensitivity was better in the older children when compared to neonates. Table 4 shows the breakdown by age group. The IRTT showed a specificity that ranged from 91.1% to 100% and a positive predictive value that ranged from 89.2% to 100%.

**Table 4:** Sensitivity, specificity, positive and negative predictive values of the tympanic thermometer.

Age group(months)	Sensitivity(%)	Specificity(%)	PPV (%)	NPV (%)
< 1	73.3	91.1	89.2	77.4
1 - 11	91.2	100.0	100.0	91.9
12 - 23	83.3	97.6	97.2	85.4
24 - 35	83.3	97.7	96.2	85.3
36 - 47	92.7	92.7	92.7	92.7
48 - 59	97.6	95.2	95.3	97.6

0 - 59	87.3	96.5	96.1	88.5

PPV: Positive predictive value

NPV: Negative predictive value

## Discussion

Oral temperatures measured using other thermometers have been found by several authors to have no significant difference with rectal (core) temperature [5,6,19]. But, in this study, the mean temperature taken with the infrared tympanic thermometer in the oral mode was significantly lower than the mean temperature taken with the rectal mercury-in-glass thermometer. The mean difference was 0.41 ± 0.37°C (p = 0.000) in the febrile group and 0.47 ± 0.39°C (p = 0.000) in the afebrile group. This may suggest that the estimation of oral temperature using the infrared tympanic thermometer in oral mode is likely to give a significantly lower temperature than the actual oral temperature.

Petersen-Smith et al [20] compared the same brand of infrared tympanic thermometer (First temp^®^) in rectal and oral modes with mercury thermometer using the same group of 232 children aged 0 - 33 months and obtained a mean temperature difference (rectal minus tympanic) of 0.05°C (-1.28 to 1.38) and 0.47°C (-0.82 to 1.76) for rectal and oral modes respectively. They concluded that the device cannot be recommended in this age group. In the present study, the infrared tympanic thermometer demonstrated no significant difference in the febrile neonates, unlike in the other age groups, though the sensitivity was poorest in that group. This may be as a result of the lower number of subjects in that group. Craig et al [21], in a meta analysis of 31 studies comprising 4441 children found that tympanic thermometer was more likely to give a lower reading than rectal thermometer, with a pooled mean difference (rectal minus tympanic) of 0.29°C. The authors also found that there was still a significant difference with rectal temperatures in all the modes (oral, rectal, core or actual mode), when analyzed separately. Oral mode gave a mean temperature difference of 0.34°C, but only two studies were included in the analysis. They also found no association between the age, the underlying temperature and the temperature difference. However, there was significant heterogeneity among the studies analyzed which might have affected the results. For instance, there was no differentiation between electronic and mercury thermometers. Also, some authors included children with otitis media in their studies.

In the present study, the correlation between the temperatures measured with the IRTT in oral mode and the rectal mercury in glass thermometer was good. It was much stronger in the febrile group than the afebrile group. Correlation, however, only demonstrates a linear relationship between two variables [22], and by implication here, as the rectal temperature increased, the tympanic temperature increased.

Despite the statistically significant difference between the mean rectal and mean tympanic temperatures, the IRTT demonstrated a fairly good sensitivity. Overall, using the IRTT in oral mode means that about 12.7% of febrile children may be missed. But, when the infrared tympanic thermometer is used in children from 3 years of age and above, the proportion of febrile children that might be missed will be 7.3% and with children ≥ 4 years, the proportion of children that might be missed will be reduced further to 2.4%. This may signify a better accuracy with increasing age and may be related to the fact that there is comparably wider ear canal with age as the thermometer probe was able to focus on the tympanum better than in the younger children. The larger number of infants in the study may have contributed to the high sensitivity in that group. In the neonates, the IRTT demonstrated relatively poor sensitivity and specificity and therefore may not be very reliable in fever detection.

The IRTT used in oral mode may not be very reliable in estimating oral temperature in children under the age of five years, but, with a sensitivity of 87.3% and a specificity of 96.5%, as well its other advantages such as speed, safety, convenience and tolerability, the infrared tympanic thermometer may be considered more attractive for fever screening in a busy paediatric hospital. Lanham et al [23] in a study in the United States of America noted that a sensitivity of 80% and specificity of 85% was too poor to continue the use of the IRTT. But, for a busy clinic or an emergency room, the speed of the instrument offers a good advantage. Monitoring of temperature can thus be done more frequently since it takes only a few seconds to measure a child's temperature, unlike the traditional mercury-in-glass thermometer, which requires a longer time to equilibrate. This makes for a more efficient decision-taking in clinical practice. However, the stated advantages of IRTT cannot be extended to neonates thus rectal thermometry should still be considered in that age group.

## Conclusions

The IRTT used in oral mode may not be reliable in estimating 'core' body temperature in children under the age of five years, but with a fairly good sensitivity and specificity, as well as its other advantages like the short duration of measurement, convenience and safety, it can still be a useful instrument for fever screening in a busy setup.

## Competing interests

The authors declare that they have no competing interests.

## Authors' contributions

BOE participated in the design, collected samples and also participated in the analysis of data and discussion. NCO conceived of the study and participated in the design, coordinated the sample collection and reviewed the results and discussion. ANI participated in the design, analysis of data and discussions.

All authors read and approved all the manuscript.
